# Predictive value of the Framingham steatosis index for cardiovascular risk: a nationwide population-based cohort study

**DOI:** 10.3389/fcvm.2023.1163052

**Published:** 2023-07-18

**Authors:** Yun Kyung Cho, Myungjin Kim, Ye-Jee Kim, Chang Hee Jung, Woo Je Lee, Joong-Yeol Park

**Affiliations:** ^1^ Department of Internal Medicine, Asan Medical Center, University of Ulsan College of Medicine, Seoul, Republic of Korea; ^2^ Asan Diabetes Center, Asan Medical Center, Seoul, Republic of Korea; ^3^ Department of Clinical Epidemiology and Biostatistics, Asan Medical Center, University of Ulsan College of Medicine, Seoul, Republic of Korea

**Keywords:** cardiovascular disease risk, cardiovascular mortality, Framingham steatosis index, non-alcoholic fatty liver disease, diabetes and endocrine research

## Abstract

**Background:**

Non-alcoholic fatty liver disease (NAFLD) is common and is associated with cardiovascular (CV) disease and mortality. The Framingham steatosis index (FSI) was recently proposed as a diagnostic marker of NAFLD and was calculated from age, body mass index, triglyceride, aspartate aminotransferase, alanine aminotransferase, diabetes history, and hypertension status. We aimed to evaluate the predictive ability of FSI for CV risk using a large-scale population dataset from the Korean National Health Insurance Service–National Health Screening Cohort (NHIS–HEALS).

**Methods:**

Among 514,866 individuals in the NHIS–HEALS, we excluded those who died, had a history of admission due to a CV event, and were heavy drinkers. The final study cohort comprised 283,427 participants. We employed both unadjusted and covariate-adjusted models in Cox proportional hazards regression analyses to determine the association between FSI and major adverse cardiovascular events (MACEs), CV events, and CV mortality.

**Results:**

During a median follow-up of 5.9 years, we documented 9,674, 8,798, and 1,602 cases of MACEs, CV events, and CV mortality, respectively. The incidence of MACEs was 1.28%, 2.99%, 3.94%, and 4.82% in the first to fourth quartiles of FSI, respectively. The adjusted hazard ratios (95% confidence interval) for MACEs gradually and significantly increased with the FSI quartiles [1.302 (1.215–1.395) in Q2, 1.487 (1.390–1.590) in Q3, and 1.792 (1.680–1.911) in Q4], following an adjustment for conventional CV risk factors, including age, sex, smoking, drinking, physical activities, low-density lipoprotein cholesterol, estimated glomerular filtration rate, and waist circumference. Participants in the higher quartiles of FSI exhibited a noteworthy increase in the occurrence of CV event. However, upon adjusting for relevant risk factors, the association between FSI and CV mortality did not reach statistical significance.

**Conclusion:**

Our study suggests that the FSI, which is a surrogate marker of NAFLD, has a prognostic value for detecting individuals at higher risk of CV events.

## 1. Introduction

Globally, cardiovascular diseases (CVDs), including ischemic heart disease (IHD) and stroke, are leading causes of mortality and morbidity (1). Since 1990, the prevalence of CVD has nearly doubled from 271 million to 525 million (1). During the same period, the number of CVD-related fatalities climbed from 12.1 to 18.6 million. In 2016, CVD accounted for 21.5% of all fatalities in Korea, only second to malignancies (28.5%). However, in terms of disability-adjusted life years, the effect of CVD was significantly greater than that of neoplasms, showing that CVDs place a huge cost burden on healthcare (2, 3). Multiple risk factors for CVD have been identified, including type 2 diabetes mellitus (T2DM) (4), obesity, hypertension, and dyslipidemia (5, 6). Most recommendations (7–9) argue for an individualized approach based on proven CV risk indicators employed in risk stratification models. However, in clinical practice, doctors frequently encounter patients with unexpected CVD events who have been incorrectly diagnosed using models based on conventional CV risk indicators. This requires accurate CVD risk estimations for the general population.

In recent years, researchers have identified non-alcoholic fatty liver disease (NAFLD) as a substantial risk factor for CVD (10). Increasing data suggest that individuals with NAFLD are at high risk of developing hypertension, coronary heart disease, cardiomyopathy, and cardiac arrhythmias, all of which are associated with increased CV morbidity and mortality (10). The histological spectrum of NAFLD ranges from simple steatosis, usually considered rather benign, to non-alcoholic steatohepatitis (NASH), which is more likely to progress to advanced fibrosis and poorer outcomes. Furthermore, NAFLD is heterogeneous with multiple etiologies and diverse histological phenotypes; particularly, inherited factors such as genes involved in lipid biology, including PNPLA3, TM6SF2, GCKR, MBOAT7, and HSD17B13, contribute to NAFLD (11). Impairment of glucose and lipid metabolic pathways, which has been typically associated with obesity and T2DM, also affect major pathological mechanistic pathways in NAFLD and its diverse metabolic fate (12).

Earlier research demonstrated that liver fibrosis, which is defined by the high fibrosis-4 (FIB-4) score or NAFLD fibrosis score, would predict increased mortality (13, 14). Framingham steatosis index (FSI) employing alanine aminotransferase (ALT)/aspartate aminotransferase (AST) ratio was proposed by Long et al. as a diagnostic measure for NAFLD in a cross-sectional analysis of 1,181 participants of the Framingham Heart Study (FHS) Third Generation Cohort (15). In the FHS cohort, the FSI model demonstrated a C-statistic of 0.830 for detecting NAFLD as identified by computed tomography. At a cut point of −1.2, the FSI exhibited a sensitivity of 79% and specificity of 71% for identifying hepatic steatosis in the FHS cohort. Similar results were observed in the third National Health and Nutrition Examination Survey (NHANES III) cohort sample at the same cut point, although with a lower sensitivity of 62% and higher specificity of 80% for detecting hepatic steatosis (15). In another recent cohort study involving 4,670 people in whom NAFLD was identified via ultrasonography, a good capacity to identify NAFLD with FSI was observed (16). The areas under the curve (AUCs) for the discriminatory and predictive abilities of the FSI in relation to NAFLD were calculated as 0.8421 [95% confidence interval (CI): 0.8314–0.8527] for the prevalence of NAFLD and 0.7093 (95% CI: 0.6863–0.7322) for the incidence of new cases of NAFLD (16). These findings demonstrate the robust performance of the FSI in both diagnostic and predictive capacities for NAFLD.

However, to date, no research has explored the ability of FSI to predict the incidence and mortality of CVD. Therefore, we sought to determine the predictive value of the FSI for major adverse cardiovascular events (MACEs) in Korean national health screening participants. In addition, we aimed to identify the subpopulation for which the FSI had a good predictive value for CV risk.

## 2. Methods

### 2.1. Study population

**Figure 1:**
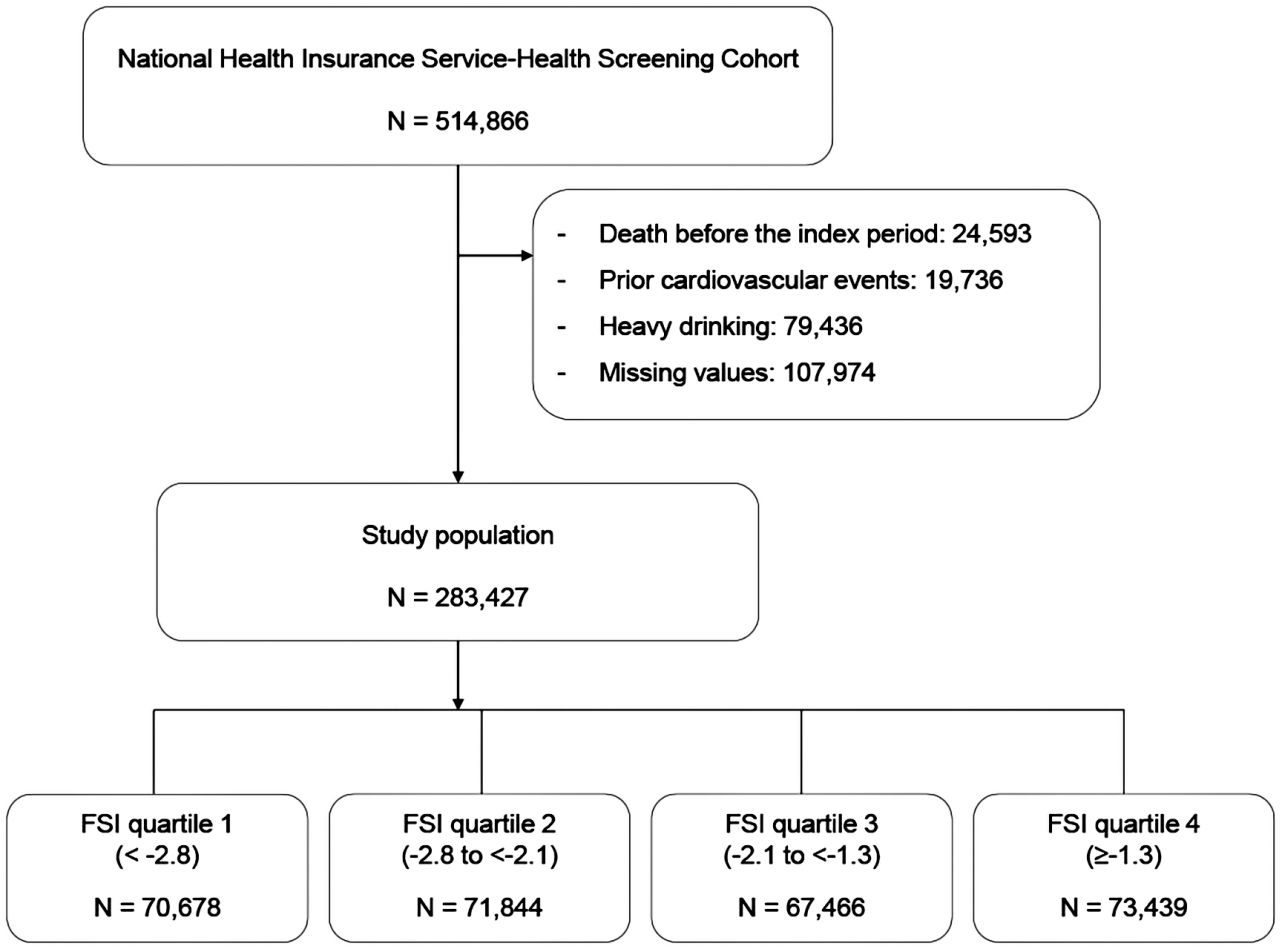
Study population.

We utilized the National Health Insurance–National Health Screening Cohort (NHIS–HEALS) data from 2002 to 2015, which was provided by the Korean NHIS in 2017. The structure and function of the Korean NHIS–HEALS are previously detailed (17). Briefly, NHIS–HEALS is a cohort of participants who participated in health screening programs provided by the NHIS in the Republic of Korea (17). The NHIS constructed the NHIS–HEALS cohort database in 2015 (17). The purpose of this cohort is to offer relevant and useful data for health researchers, especially in the field of non-communicable diseases and health risk factors, and policymaker (17). Recently, numerous researchers in medical fields have been conducting studies using this cohort. The period from 10 January 2009 to 31 December 2010 was chosen as the index period because of the presence of the required lipid profiles to define metabolic health in the NHIS–HEALS since 2009 (17). Patients who died or had a history of hospitalization due to a CV event before the end of the index period were excluded from the analysis of the 514,866 participants in the NHIS–HEALS. After this exclusion, 362,863 patients were included in our earlier investigation using these data (18). In the present study, we additionally excluded heavy drinkers who consumed seven or more drinks on each occasion and consumed alcohol more than 5 days per week. The final study cohort comprised 283,427 patients. Figure 1 displays the participants and the research design. This study was approved by the Asan Institutional Review Board (2021-0783).

### 2.2. Calculation of the FSI

The FSI was calculated based on the following formula (15):

$$\begin{array}{r} \mathrm{FSI}=\;-\;7.981+0.011\times \mathrm{age}\;(\mathrm{years})\\ -\;0.146\times \mathrm{sex}\;(\mathrm{female}=1,\;\;\mathrm{male}=0)\\ +\;0.173\times \mathrm{BMI}\;(\mathrm{kg}/m^{2})+0.007\times \mathrm{triglycerides}\;(\mathrm{mg}/\mathrm{dl})\\ +\;0.593\times \mathrm{hypertension}\;(\mathrm{yes}=1,\;\;\mathrm{no}=0)\\ +\;0.789\times \mathrm{diabetes}\;(\mathrm{yes}=1,\;\;\mathrm{no}=0)\\ +\;1.1\times \mathrm{ALT}:\mathrm{AST}\;\mathrm{ratio}\geq 1.33\;(\mathrm{yes}=1,\;\;\mathrm{no}=0) \end{array}$$

Subsequently, we classified the study population into four groups according to the FSI quartiles (Q1, <−2.8; Q2, −2.8 to <−2.1; Q3, −2.1 to <−1.3; and Q4, ≥−1.3) (15).

### 2.3. Definition of outcomes

MACE, a composite of non-fatal myocardial infarction (MI), non-fatal stroke, and cardiovascular death, was the main outcome. Admissions due to MI and stroke (ischemic or hemorrhagic) were classified as CV events between 1 January 2011 and 31 December 2015. Occurrence of the CV events was detected using the hospital discharge data. We included individuals with MI or stroke as codes for a primary or secondary condition in the 10th revision of the International Classification of Diseases (ICD-10). CV mortality was defined as death caused by circulatory system disorders (I00-99).

### 2.4. Definitions of type 2 diabetes, hypertension, and dyslipidemia

Prescription of antidiabetic drugs and reporting of ICD-10 codes E11 (non-insulin-dependent diabetes mellitus), E12 (malnutrition-related diabetes mellitus), E13 (other specified diabetes mellitus), and E14 (unspecified diabetes mellitus) as the primary or secondary diagnoses were used to define T2DM. During the study period, pharmacies in Korea supplied eight different types of diabetic medications: sulfonylureas, biguanides, glucosidase inhibitors, thiazolidinediones, meglitinide, glucagon-like peptide-1 receptor agonists, dipeptidyl peptidase-4 inhibitors, and insulin.

Participants whose primary or secondary diagnoses were I10 (primary hypertension), I11 (hypertensive heart disease), I12 (hypertensive chronic kidney disease), I13 (hypertensive heart and chronic kidney disease), or I15 (secondary hypertension) were classed as having hypertension. Angiotensin receptor blockers, angiotensin-converting enzyme inhibitors, beta blockers, calcium channel blockers, and diuretics were administered by pharmacies during the study period. The use of lipid-lowering medications and reporting of ICD-10 code E78 (disorders of lipoprotein metabolism and other lipidemias) as the primary or secondary diagnosis constituted dyslipidemia. Lipid-lowering medications included statins, ezetimibe, and fibrates.

### 2.5. Covariates

Covariates from the baseline health examination included smoking status (non-smoker, ex-smoker, or current smoker), drinking status (none, light, or moderate drinking), physical activity (zero, one to two, three to four, or five times per week), low-density lipoprotein cholesterol (LDL-C) level, estimated glomerular filtration rate (eGFR), and waist circumference. Light and moderate drinkers consumed seven drinks per day and drank 1–2 or 3–4 days per week, respectively.

### 2.6. Statistical analysis

Using Cox proportional hazards models, we evaluated the hazard ratios (HRs) and 95% CIs for MACEs, CV events, and CV mortality. Multivariate models were adjusted for smoking, drinking, physical activity, eGFR, LDL-C levels, and waist circumference; age, sex, and BMI were not adjusted because the FSI was calculated using these data. The reference group consisted of individuals with the lowest FSI quartile in the whole cohort or in each subgroup analyses. The SAS Enterprise Guide (version 7.1, SAS Institute Inc., Cary, NC, United States) and R version 4.3.0 from the R Project for Statistical Computing was used for all statistical analyses.

## 3. Results

### 3.1. Baseline characteristics of the study cohort

Table 1 summarizes the baseline clinical and biochemical features of the individuals according to their FSI quartile. FSI was linked with atherosclerotic CVD (ASCVD) risk factors and metabolic syndrome components, including current smoking, total cholesterol, LDL-C, and the prevalence of dyslipidemia (all *p* < 0.001). Those with a higher FSI engaged in less physical activity (all *p* < 0.001). The FSI was negatively correlated with eGFR (all *p* < 0.001).

**Table 1:** Characteristics of study participants according to the Framingham steatosis index quartiles.

	Q1 (<−2.8)	Q2 (−2.8 to −2.1)	Q3 (−2.1 to −1.3)	Q4 (−1.3 ≤)	*p*-value
N	70,678	71,844	67,466	73,439	
Sex (% men)	32.3	42.3	46.7	53.8	<0.0001
Age (years)	56.6 ± 8.2	59.2 ± 8.7	61.1 ± 8.9	61.1 ± 8.9	<0.0001
BMI (kg/m^2^)	21.3 ± 1.9	23.5 ± 1.9	24.8 ± 2.2	26.4 ± 2.8	<0.0001
WC (cm)	74.2 ± 6.2	80.3 ± 6.3	83.8 ± 6.5	88.1 ± 7.4	<0.0001
Systolic BP (mmHg)	118.9 ± 13.9	124.6 ± 14.5	128.5 ± 14.9	131.2 ± 15	<0.0001
Diastolic BP (mmHg)	74 ± 9.3	77.2 ± 9.6	79.1 ± 9.8	80.7 ± 9.8	<0.0001
Smoking (%)					<0.0001
Current smoker	9.8	14.3	16.6	19.5	
Ex-smoker	10.0	11.5	12.1	15.0	
Non-smoker	80.2	74.2	71.3	65.5	
Drinking (%)					<0.0001
None	74.6	73.5	68.9	74.3	
Mild	20.9	22.5	20.8	23.2	
Moderate	4.4	5.6	5.8	6.4	
Physical activity (%)					<0.0001
None	27.9	29.2	29.6	31.3	
1–2 times/week	22.2	21.6	21.2	22.7	
3–4 times/week	21.9	21.7	21.2	20.6	
≥5 times/week	27.8	27.5	27.9	25.3	
Hypertension (%)	4.9	24.5	49.3	65.1	<0.0001
Diabetes (%)	0.5	2.8	9.6	29.6	<0.0001
Dyslipidemia (%)	8.1	15.6	25.3	37.0	<0.0001
FPG (mg/dl)	93.5 ± 13.9	97 ± 17.3	101.7 ± 23.1	112.9 ± 35.1	<0.0001
TG (mg/dl)	83 ± 29.5	113.6 ± 40.9	142 ± 55.7	215.7 ± 119.9	<0.0001
HDL-C (mg/dl)	59.5 ± 20.1	54.8 ± 23	52 ± 24.1	49.5 ± 35.2	<0.0001
LDL-C (mg/dl)	120.8 ± 35	126 ± 36	125.2 ± 37.8	118.5 ± 44.2	<0.0001
TC (mg/dl)	197.2 ± 34.5	203.1 ± 36.3	204.7 ± 38.1	207.9 ± 41	<0.0001
AST (U/L)*	23 (19–27)	23 (20–28)	24 (20–29)	25 (21–32)	<0.0001
ALT (U/L)*	17 (14–22)	19 (15–25)	22 (17–29)	28 (20–40)	<0.0001
ALT/AST ratio	0.77 (0.65–0.92)	0.84 (0.70–1.00)	0.91 (0.75–1.10)	1.11 (0.87–1.42)	<.0001
eGFR (mL/min/1.73 m^2^)	83.5 ± 18.4	80.3 ± 18.6	78.2 ± 18.8	76.5 ± 19.7	<0.0001

Results reported as means ± SD or percentage, unless otherwise indicated.

* Median (interquartile range).

BP, blood pressure; FPG, fasting plasma glucose; GGT, gamma-glutamyl transpeptidase; HDL-C, high-density lipoprotein cholesterol; TC, total cholesterol; TG, triglyceride; WC, waist circumference.

### 3.2. MACEs, CV events, and CV mortality according to the FSI quartiles

**Figure 2:**
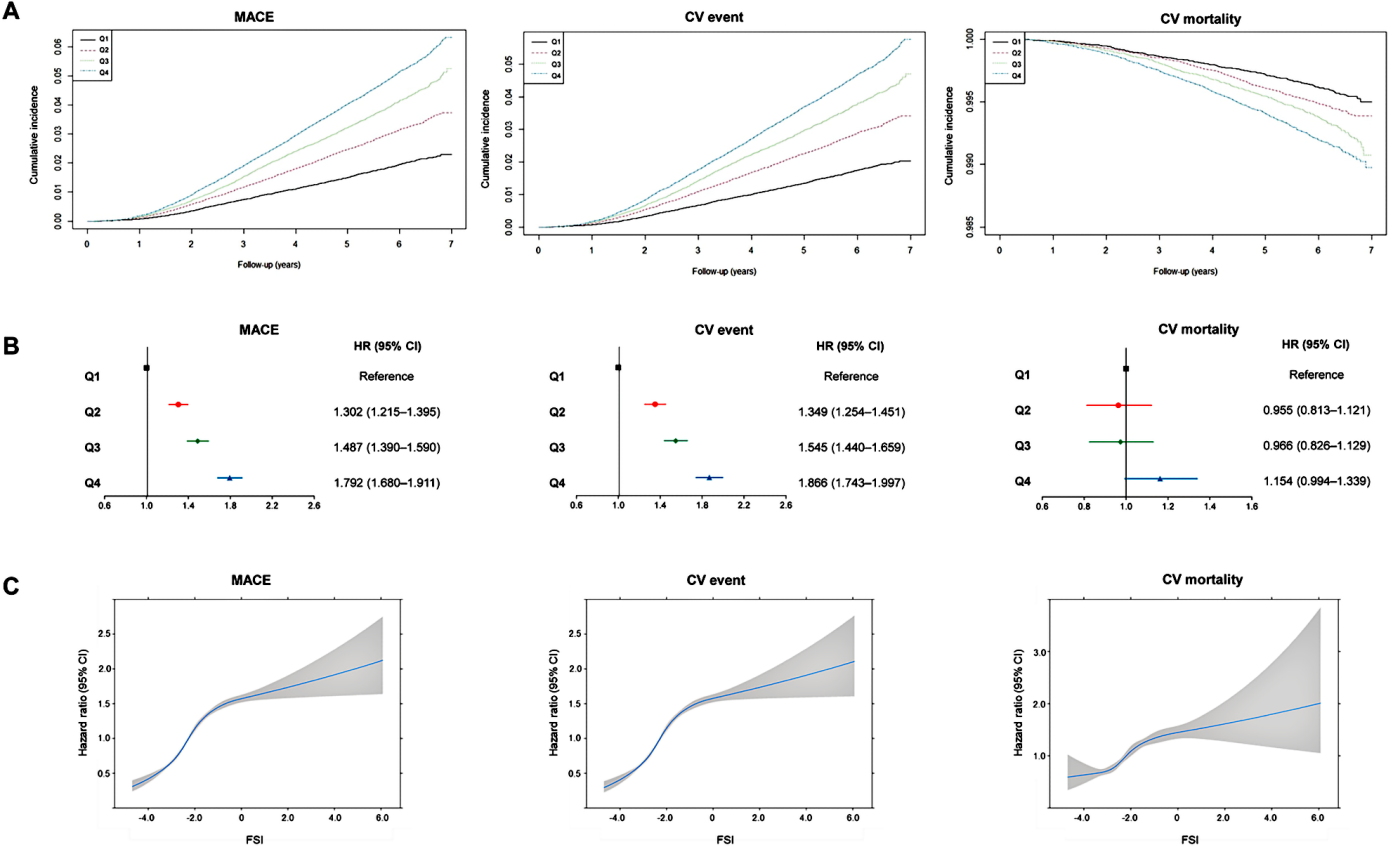
Association between Framingham steatosis index and cardiovascular outcomes. (**A**) Cumulative incidence of major adverse cardiovascular events, cardiovascular events, and cardiovascular mortality according to Framingham steatosis index quartiles. (**B**) Summarized figure of HRs for major adverse cardiovascular events, cardiovascular events, and cardiovascular mortality according to Framingham steatosis index quartiles. (**C**) Cubic spline graph of hazard ratio (blue line) and 95% confidence interval (gray-shaded area) (*y*-axis) relative to FSI (*x*-axis) for major adverse cardiovascular events, cardiovascular events, and cardiovascular mortality.

We observed 9,674, 8,798, and 1,602 instances of MACEs, CV events, and CV deaths, respectively, in 283,427 individuals. The cumulative incidence of MACEs, CV events, and CV mortality are shown in Figure 2A. The highest quartile of the FSI was related to the highest risk of MACEs, CV events, and CV mortality. In lower quartiles, the risks for MACEs and CV events progressively declined (log-rank *p* < 0.001).

During the follow-up period, 3,564 patients with the greatest FSI (Q4) experienced MACEs (overall incidence, 4.82%), whereas only 1,308 patients with the lowest FSI (Q1) reported less experience of MACEs (overall incidence, 0.46%) (Table 2). Crude HRs for MACEs increased for the second (1.613, 95% CI: 1.506–1.728), third (2.128, 95% CI: 1.991–2.273), and fourth (2.634, 95% CI: 2.472–2.806) FSI quartiles relative to the first quartile (Table 2). HRs for CV events increased as follows: 1.654 (95% CI: 1.538–1.778), 2.170 (95% CI: 2.024–2.327), and 2.688 (95% CI: 2.514–2.874) for the second, third, and fourth quartiles, respectively, compared with the first quartile (Table 2). Similarly, CV mortality considerably increased among individuals in the higher FSI quartiles, i.e., 1.328 (95% CI: 1.132–1.558), 1.661 (95% CI: 1.423–1.939), and 2.065 (95% CI: 1.783–2.391) for the second, third, and fourth quartiles, compared with the first quartile (Table 2). The covariate-adjusted model for age, sex, smoking, alcohol consumption, physical activity, LDL cholesterol, eGFR, and waist circumference still revealed a significant and progressive increase in the risk of MACEs and CV events [HR (95% CI) for MACEs from second to fourth FSI quartiles: 1.302 (1.215–1.395), 1.487 (1.390–1.590), and 1.792 (1.680–1.911); HR (95% CI) for CV events: 1.349 (1.254–1.451), 1.545 (1.440–1.659), and 1.866 (1.743–1.997)]. However, when it comes to CV mortality, the FSI was not significantly associated with CV mortality after adjusting for covariates [HR (95% CI) for CV mortality: 0.955 (0.813–1.121), 0.966 (0.826–1.129), and1.154 (0.994–1.339)] (Table 2). Figure 2B presents the adjusted HRs for MACEs, CV events, and CV mortality by FSI quartile.

**Table 2:** Hazard ratios for (A) major adverse cardiovascular events, (B) cardiovascular events, and (C) cardiovascular mortality according to the Framingham steatosis index quartiles.

		Event *N* (%)	Unadjusted	Adjusted
(A) MACEs	Q1	1,308 (1.28)	Reference	Reference
	Q2	2,146 (2.99)	1.613 (1.506–1.728)	1.302 (1.215–1.395)
	Q3	2,656 (3.94)	2.128 (1.991–2.273)	1.487 (1.390–1.590)
	Q4	3,564 (4.82)	2.634 (2.472–2.806)	1.792 (1.680–1.911)
(B) Cardiovascular events	Q1	1,168 (1.65)	Reference	Reference
	Q2	1,964 (2.73)	1.654 (1.538–1.778)	1.349 (1.254–1.451)
	Q3	2,419 (3.59)	2.170 (2.024–2.327)	1.545 (1.440–1.659)
	Q4	3,247 (4.42)	2.688 (2.514–2.874)	1.866 (1.743–1.997)
(C) Cardiovascular mortality	Q1	261 (0.37)	Reference	Reference
	Q2	355 (0.49)	1.328 (1.132–1.558)	0.955 (0.813–1.121)
	Q3	419 (0.62)	1.661 (1.423–1.939)	0.966 (0.826–1.129)
	Q4	567 (0.77)	2.065 (1.783–2.391)	1.154 (0.994–1.339)

Q, quartile.

Adjusted HRs (95% CIs) were adjusted for age, sex, smoking, alcohol drinking, physical activities, LDL cholesterol, eGFR levels, and waist circumference.

A sensitivity analysis was conducted to examine the hazard ratios using the FSI as continuous data. The results indicated a gradual increase in the risk of MACE, CV events, and CV mortality as the FSI values increased (Figure 2C). The HRs for MI and stroke demonstrated a positive association with increasing values of the FSI; the multivariable-adjusted HRs (95% CI) for the second to fourth quartiles were 1.457 (1.178–1.801), 2.102 (1.722–2.567), and 3.092 (2.559–3.736) for MI and 1.290 (1.194–1.394), 1.417 (1.314–1.528), and 1.593 (1.481–1.713), respectively (Supplementary Table S1 in the Supplementary Appendix). When we employed the FSI cutoff of −1.2 to define NAFLD, participants with an FSI higher than this cutoff exhibited a significantly increased CV risk, and these associations remained significant even after adjusting for other covariates [HR (95% CI): 1.387 (1.329–1.448), 1.402 (1.340–1.466), 1.204 (1.083–1.338), 1.956 (1.758–2.176), and 1.263 (1.203–1.326) for MACE, CV events, CV mortality, MI, and stroke, respectively) (Supplementary Table S2 in the Supplementary Appendix).

### 3.3. Subgroup analyses

**Figure 3:**
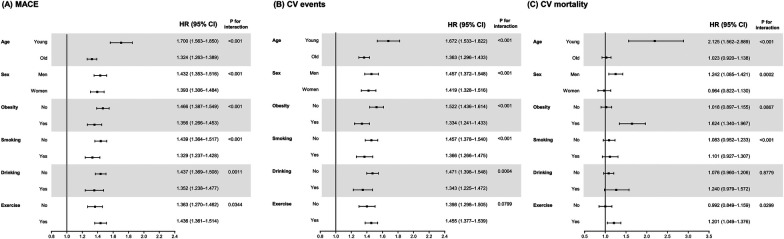
Subgroup analyses for the risk of (**A**) major adverse cardiovascular events, (**B**) cardiovascular events, and (**C**) cardiovascular mortality according to Framingham steatosis index quartiles. The high (upper half) and low (lower half) FSI groups have been compared after adjusting for smoking, alcohol drinking, physical activities, LDL cholesterol, and eGFR levels. The covariates are excluded from the adjustment in the corresponding subgroup analyses.

Next, we calculated the adjusted HRs of outcomes comparing high FSI (Q3 and Q4) vs. low FSI (Q1 and Q2) in subgroups according to age, sex, the presence of obesity, smoking status, drinking, and physical activity (Figure 3). The predictive value of FSI was especially evident when the participants are young [HR (95% CI) for MACEs in high FSI: 1.700 (1.563–1.850) in young population and 1.324 (1.263–1.389) in old population, *p*-value for interaction of <0.001]. Particularly, when it comes to CV mortality, high FSI was significantly associated with increased risk in young population [HR (95% CI): 2.125 (1.562–2.889)] and obese population [1.624 (1.340–1.967)].

## 4. Discussion

### 4.1. Principal results of this study

**Figure 4:**
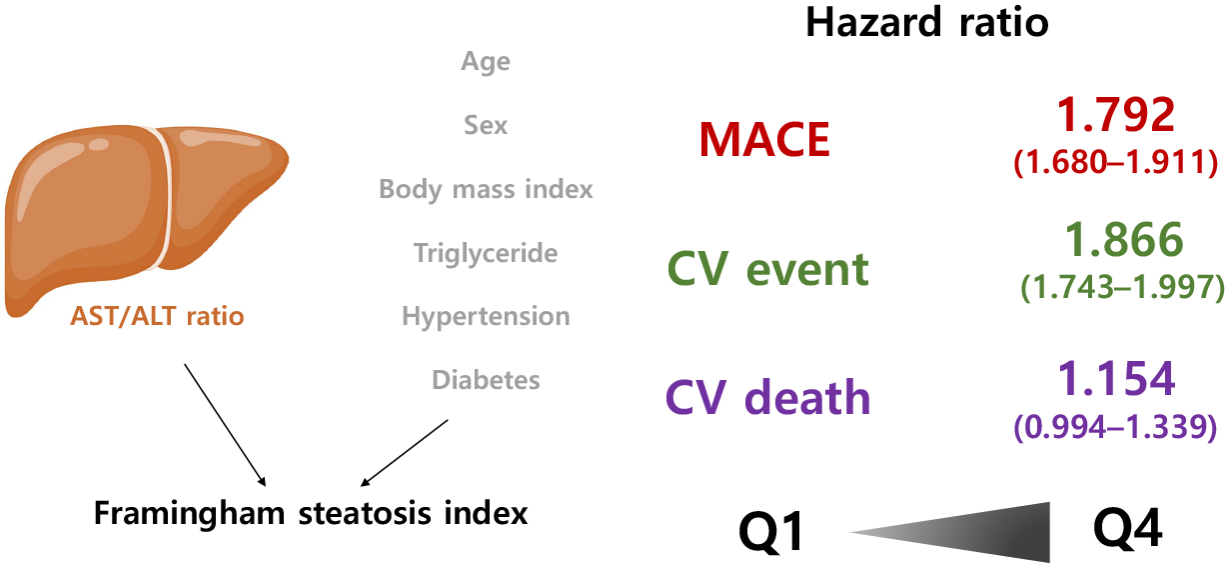
Summarized figure of the main finding of the study. Q, quartile.

Using a large-scale, nationwide cohort dataset, we studied the association between FSI and CV risk. Even after adjusting for potential confounding variables such as CV risk factors, a high FSI was associated with a substantially increased risk of future MACEs (Figure 4). In the subgroup analysis based on baseline characteristics, we consistently identified the predictive value of FSI for MACEs, demonstrating the persistence of this relationship. Subgroup analyses revealed that the predictive value of FSI for CV risk is outstanding in young individuals. Observational studies should be interpreted precaution; however, this large, nationwide observational study, which includes more than 280,000 individuals, revealed that a high FSI is a significant predictor of future CV events. This is the largest and first ever study to explore the association between FSI, a clinical marker of NAFLD, and future CV events in the general population.

### 4.2. Known association between NAFLD and CV risk

Several research studies have indicated that NAFLD is an underappreciated and independent risk factor for ASCVD, even when controlling for ASCVD risk factor factors (19–25). Accumulating evidence indicates a close association between NAFLD and an elevated risk of CV events and mortality. Individuals with NAFLD often exhibit a higher prevalence of traditional CV risk factors, including obesity, insulin resistance, dyslipidemia, and hypertension. Notably, NAFLD has been identified as an independent predictor of adverse CV outcomes, such as MI and stroke, as supported by our own analyses. In a comprehensive meta-analysis published in 2021, a significant association was observed between the presence of NAFLD and fatal CVD events [10 studies; pooled random effects (HR 1.30, 95% CI: 1.08–1.56)]. Furthermore, NAFLD was found to be associated with an increased risk of non-fatal CVD events alone (13 studies; HR 1.40, 95% CI: 1.20–1.64), as well as an increased risk of both fatal and non-fatal CVD events combined (10 studies; HR 1.81, 95% CI: 1.39–2.36) (26).

The association between NAFLD and CVD has been consistently reported in the literature. However, when examining the specific outcome of CVD mortality in individuals with NAFLD, conflicting results have been observed across various studies. In a previous meta-analysis incorporating eight longitudinal cohort studies (27), Wu et al. found that NAFLD was associated with a nearly 40% increased risk of non-fatal CVD events [three studies; pooled random effects (HR 1.37, 95% CI: 1.10–1.72)]. However, the association between NAFLD and CVD mortality was not statistically significant [five studies; random effects (HR 1.10, 95% CI: 0.86–1.41)]. Similarly, another meta-analysis including seven cohort studies (28) conducted by Liu et al. reported that NAFLD was significantly associated with increased all-cause mortality but did not significantly predict CVD mortality [pooled random effects (HR 1.13, 95% CI: 0.92–1.38)]. These discrepancies in findings could be attributed to variations in the definition and severity of NAFLD, differences in study populations, and the choice of covariates used in the analyses. In our own analyses, we observed a significantly higher cumulative incidence of CVD mortality in the higher FSI groups, and the crude hazard ratios also demonstrated an increasing trend with higher FSI index. However, when adjusting for covariates, the FSI index was no longer predictive of CVD mortality. Given that mortality is influenced by multiple factors and study design can impact results, further research is warranted to gain a better understanding of this complex issue.

A recent meta-analysis provided interesting insights into the association between NAFLD and cardiovascular outcomes. The study reported that patients with NAFLD have a higher risk of atrial fibrillation (AF), heart failure (HF), MI, and stroke compared with individuals without NAFLD (29). While the precise mechanisms underlying the relationship between NAFLD, HF, and AF are not fully elucidated, NAFLD has been associated with cardiac remodeling, which has the potential to contribute to the development of HF and AF (30–33). Further epidemiological studies are needed to better corroborate the association between NAFLD and HF and AF. Interestingly, the aforementioned meta-analysis also identified a noteworthy inverse association between mean age and odds ratios for stroke at the univariable level in meta-regressions to confirm the importance of study-level characteristics (29). This suggests that other variables may modulate the risk in NAFLD patients in elderly patients, and the impact of NAFLD on cardiovascular events may be more pronounced in younger cohorts. These findings align with our subgroup analyses, which demonstrated the robust predictive value of the FSI for CVD in the young population. Although the underlying mechanisms for this observation were not explored in the current epidemiological study, the differential impact of NAFLD across different age groups holds a significant value and warrants further investigation.

The underlying risk factors for NAFLD, such as dyslipidemia and dysregulation of glucose homeostasis, contribute to the elevated risk of ASCVD in NAFLD. However, the predilection for ectopic fat deposition in the liver and other tissues appears to be associated with an elevated risk of ASCVD above and beyond the risk attributable to traditional risk factors. Endothelial dysfunction and higher systemic inflammation are also associated with NAFLD (34, 35) in addition to the aforementioned risk factors, including ectopic fat accumulation in other organs (e.g., the pancreas, skeletal muscle, and epicardium) (36). Aberrant systemic lipid metabolism significantly contributes to the development of NAFLD (37). When insulin resistance is present, the visceral adipose tissue exhibits metabolic dysregulation. Hormone-sensitive lipase is no longer properly controlled by insulin within adipocytes, resulting in increased lipolysis of adipocyte triglycerides and levels of free fatty acids in the circulation (38), and then, insulin resistance exacerbates the predisposition for increased liver fat accumulation (39). Therefore, it is difficult to identify the specific contribution of NAFLD to increased CVD risk, particularly in clinical studies, because NAFLD and CVD share several risk factors including insulin resistance as described above (40). However, growing evidence suggests that NAFLD is an independent risk factor for CVD. In addition to metabolic dysregulation such as insulin resistance, various hepatokines associated with the liver–gut axis can induce endothelial cell deterioration through inflammatory reactions and oxidative stress, structural changes in blood vessels, and modifications in blood coagulation factors (41). For example, the first hepatokine of which role in metabolic diseases was revealed is *α*2-HS-glycoprotein (fetuin-A). Production of this glycoprotein is increased in steatotic and inflamed liver and has a major role in the pathophysiology of T2DM and CVD in humans (42). Although these mechanisms presumably link NAFLD to the onset and progression of CVD, there is a lack of studies on the relationship between these two entities.

### 4.3. FSI is an accurate indicator of future CV adverse outcome, as well as NAFLD

In 2016, Long et al. found that in a large cross-sectional investigation of community-based Framingham Heart Study participants who underwent abdominal computed tomography scans, the ALT/AST ratio was more indicative of hepatic steatosis than ALT or AST alone. The authors developed the FSI, a simple clinical model for predicting hepatic steatosis, including BMI, lipid levels, hypertension, diabetes, and the ALT/AST ratio. FSI has been proven to distinguish individuals with NAFLD (15). Moreover, according to a second Iranian study with a total of 4,670 participants, FSI has a good potential to identify NAFLD (16). However, to date, no research has been conducted on the therapeutic relevance of FSI in terms of CV outcomes or mortality.

Our current study added the value of FSI as a surrogate for future CV event. The incidence of MACEs and CV events was gradually increased with higher FSI quartiles. Even after adjustment for other risk factors including LDL-C, the HRs for MACEs and CV events also gradually and significantly increased with the higher FSI quartiles, supporting the value of this index as a CV risk predictor. For the first time, we assessed the predictive power of FSI for CV outcomes and found that a high FSI was strongly related to an increased risk of MACEs. FSI may be a valuable surrogate diagnostic measure for hepatic steatosis in large epidemiological investigations when neither liver imaging nor liver biopsy is available. Because the FSI's constituent components are typically readily available, this approach offers a substantial improvement over existing diagnostic markers. Currently, there is a lack of clear guidelines regarding the cutoff values of FSI for the diagnosis of NAFLD. However, some available data on FSI cutoff points exist. Long et al. (15) suggested a cutoff value of −1.2 for defining patients with NAFLD. In our study, we conducted additional analyses to determine whether this cutoff value could effectively discriminate the participants at high CV risk. Our analyses revealed that participants with an FSI higher than the cutoff value of −1.2 exhibited an increased risk of MACE, CV events, CV mortality, MI, and stroke, even after adjusting for other covariates (Supplementary Table S1 in the Supplementary Appendix). These findings highlight the prognostic significance of FSI in predicting adverse cardiovascular outcomes.

Our subgroup analyses revealed that the predictive value of this index is particularly outstanding in young population. The HRs (95% CI) in the high FSI group, compared with the low FSI group, was 1.675 (1.521–1.845) for MACEs, 1.663 (1.506–1.836) for CV events, and 1.716 (1.211–2.434) for CV mortality. Our investigation revealed a noteworthy and statistically significant increase in FSI levels within the older age group in comparison with the younger age group (−2.1 ± 1.1 vs. −1.7 ± 1.1, *p* < 0.05). This finding is consistent with our expectations, as FSI, including other indices such as FIB-4 (36615725 = 40), incorporates age as a variable in its formula. The disparities in the impact of FSI on CVD risk between younger and elderly populations may be partially attributed to the variation in FSI values between the two age groups. Nevertheless, even after adjusting for age, the hazard ratio for the development of CVD significantly remained higher in the group with elevated FSI levels. Additionally, the interaction tests conducted between the subgroups revealed a *p*-value for interaction of less than 0.05, indicating that the predictive capacity of FSI is more robust in the younger population. One possible explanation for this result is that in older population, other factors such as underlying complex medical condition, which could not be included in the analyses, act as powerful confounders for the association between FSI and CV outcomes. Although our epidemiologic study could not clarify the mechanisms, clinically, our results suggest that young patients could benefit from the calculation of this index for the prediction of their CV risk.

### 4.4. Strengths and limitations

This study had some limitations. First, our findings may not be generalizable to other ethnic groups, given that we included only Korean participants. Second, a small number of events and short follow-up periods may have rendered the study insufficiently powerful to accurately evaluate relationships. Third, our definition of CV events based on the claimed data may not be entirely reliable. We defined the outcomes by merging the diagnostic and medication histories to improve precision. Even when CV risk variables were controlled, there may have been other confounding factors. Despite adjusting the analyses for the majority of the available demographic and clinical characteristics, unrevealed factors may have affected our findings. The use of the FSI instead of histological examination as a proxy measure of NAFLD was another drawback of our study. In a previous study, Higashiura et al. found that the FIB-4 index was predictive of the development of ischemic heart disease in individuals with fatty liver, but not in those without fatty liver (43). Similarly, it is possible that the predictive value of FSI is influenced by the presence of fatty liver. However, due to the unavailability of imaging study results in the NHIS data to assess fatty liver disease, we were unable to analyze the implications of FSI based on the diagnosis of fatty liver. One of the limitations of this study is the presence of competing risks, which can introduce complexities in the analysis and interpretation of the results (44). In our investigation, we focused on a CV disease and CV mortality, but it is essential to acknowledge that there may be other concurrent events that could impact the interpretation of our findings. Although CV mortality is a major cause of death, it is imperative to recognize and account for the presence of competing risks—such as non-cardiovascular deaths or other causes of mortality—when interpreting the outcomes of our study. Lastly, we did not compare the performance of the FSI with other established CV risk tests, including a 10-year ASCVD risk calculator or Framingham Risk Score, which could limit the acceptance of FSI as a standardized risk calculator in a general population. However, to circumvent this restriction, we conducted subgroup analyses based on the presence or absence of each CV risk factor, and the link between FSI and MACEs was consistently detected across subgroups. The strengths of our study were the use of a large national database and demonstration of the function of FSI in predicting future MACEs and mortality via an adjusted analysis with several confounding factors and subgroup analyses. Our results revealed that the FSI may be utilized to assess future CV risk in clinical settings at an affordable price.

To date, several surrogate indicators have been developed and proposed to predict future CV events. For instance, we have previously reported the significant association of the atherogenic index of plasma (AIP) with cardiovascular risk (18). The AIP is composed of triglycerides and high-density lipoprotein cholesterol and is a novel marker for assessing the risk of atherogenicity, which demonstrated the association with cardiometabolic risk (45–47). In the present study, we suggest the FSI as another significant marker for CV risk, by demonstrating that participants with the higher FSI quartiles had higher HRs for MACEs, CV events, and CV mortality. The distinct implication of the FSI is that this index integrates AST/ALT ratio, a laboratory measure related to fatty liver, with conventional risk factors. A notable strength of the study is that it is the first to demonstrate the predictive utility of the FSI for cardiovascular risk, while previous literatures focused on the value of the FSI as an indicator of NAFLD.

In conclusion, a higher FSI was associated with an increased risk of MACEs, CV events, and CV mortality in this large national cohort. Specifically, the FSI may be a strong predictor of future CV events in young individuals. Apart from its role in the diagnosis of NAFLD, it can be used as an effective marker of mass screening to identify people at high risk for CV events.

## Data Availability

Publicly available datasets were analyzed in this study. These data can be found here: https://nhiss.nhis.or.kr/ (NHIS-2021-2-259).
