# Supplementary material for: Predictive value of the Framingham steatosis index for cardiovascular risk: a nationwide population-based cohort study
Source: Front Cardiovasc Med. 2023 Jul 18;10:1163052. doi: 10.3389/fcvm.2023.1163052 (PMC10391153; doi:10.3389/fcvm.2023.1163052)
Supplement: Supplementary file 1 [file Table1.docx]

**Supplementary appendix**

**Table S1.** Hazard ratios for (A) Myocardial infarction, and (B) ischemic stroke according to the Framingham steatosis index quartiles.

|  |  | Event N (%) | Unadjusted | Adjusted |
| --- | --- | --- | --- | --- |
| 1. Myocardial  infarction | Q1 | 132 (0.19) | Reference | Reference |
|  | Q2 | 246 (0.34) | 1.825 (1.477–2.255) | 1.457 (1.178–1.801) |
|  | Q3 | 379 (0.56) | 2.986 (2.450–3.641) | 2.102 (1.722–2.567) |
|  | Q4 | 645 (0.88) | 4.677 (3.878–5.640) | 3.092 (2.559–3.736) |
| (B) Stroke | Q1 | 1048 (1.48) | Reference | Reference |
|  | Q2 | 1735 (2.41) | 1.534 (1.421–1.657) | 1.290 (1.194–1.394) |
|  | Q3 | 2066 (3.06) | 1.863 (1.729–2.006) | 1.417 (1.314–1.528) |
|  | Q4 | 2647 (3.60) | 2.159 (2.010–2.319) | 1.593 (1.481–1.713) |

*CI* confidence interval, *HR* hazard ratio, and *Q* quartile

Adjusted HRs (95% Cis) were adjusted for the age, sex, smoking, alcohol drinking, physical activities, LDL-cholesterol, eGFR levels, and waist circumference.

**Table S2.** Hazard ratios for (A) major adverse cardiovascular events, (B) cardiovascular events, (C) cardiovascular mortality, (D) myocardial infarction, and (E) ischemic stroke according to the Framingham steatosis index.

|  | FSI | Event N (%) | Unadjusted | Adjusted |
| --- | --- | --- | --- | --- |
| (A) MACEs | <-1.2 | 6360/216559 (2.94) | Reference | Reference |
|  | -1.2≤ | 3314/66868 (4.96) | 1.663 (1.595–1.735) | 1.387 (1.329–1.448) |
| (B) Cardiovascular events | <-1.2 | 5779/216559 (2.67) | Reference | Reference |
|  | -1.2≤ | 3020/66868 (4.52) | 1.667 (1.595–1.742) | 1.402 (1.340–1.466) |
| (C) Cardiovascular mortality | <-1.2 | 1078/216559 (0.50) | Reference | Reference |
|  | -1.2≤ | 524/66868 (0.78) | 1.550 (1.396–1.720) | 1.204 (1.083–1.338) |
| (D) Myocardial  infarction | <-1.2 | 801/216559 (0.37) | Reference | Reference |
|  | -1.2≤ | 601/66868 (0.90) | 2.421 (2.178–2.691) | 1.956 (1.758–2.176) |
| (E) Ischemic stroke | <-1.2 | 5041/216559 (2.33) | Reference | Reference |
|  | -1.2≤ | 2455/66868 (3.67) | 1.456 (1.387–1.528) | 1.263 (1.203–1.326) |

*CI* confidence interval, *HR* hazard ratio, and *Q* quartile

Adjusted HRs (95% CIs) were adjusted for the age, sex, smoking, alcohol drinking, physical activities, LDL-cholesterol, eGFR levels, and waist circumference.
